# Three lifestyle-related issues of major significance for public health among the Inuit in contemporary Greenland: a review of adverse childhood conditions, obesity, and smoking in a period of social transition

**DOI:** 10.1186/s40985-018-0085-8

**Published:** 2018-04-16

**Authors:** Peter Bjerregaard, Christina V. L. Larsen

**Affiliations:** 1 National Institute of Public Health, University of Southern Denmark, Copenhagen, Denmark; 2 Greenland Centre for Health Research, University of Greenland, Nuuk, Greenland

**Keywords:** Inuit, Greenland, Alcohol, Suicide, Diet, Obesity, Smoking

## Abstract

Greenland is a country in transition from a colonial past with subsistence hunting and fishing to an urban Nordic welfare state. Epidemiological transition from infectious to chronic diseases has been evident since the 1950s. Ninety percent of the population is Inuit.

We studied three public health issues based on published literature, namely adverse childhood experiences, addictive behavior, and suicide; diet and obesity; and smoking. Alcohol consumption was high in the 1970s and 1980s with accompanying family and social disruption. This is still a cause of poor mental health and suicides in the generations most affected. The diet is changing from a traditional diet of fish and marine mammals to imported food including food items rich in sugar and fat from domestic animals, and the level of physical activity is decreasing with an ensuing epidemic rise in obesity. The prevalence of smoking is high at around 60% among both men and women and is only slowly decreasing. Smoking shows large social variation, and tobacco-related diseases are widespread.

The diseases and conditions outlined above all contribute towards a low life expectancy at birth—69 years for men and 74 years for women in 2011–2015—compared with 78 and 84 years for men and women, respectively, on average in the European countries. The translation of government public health programs into local activities needs strengthening, and it must be realized that the improvement of public health is a long-term process.

## Background

Greenland is a self-governing part of the Kingdom of Denmark with a majority population of Inuit (ca. 90%). The traditional livelihood of the Inuit was the hunting of marine mammals, seals in particular, which necessitated a decentralized settlement pattern. Due to a warming of ocean temperatures in the beginning of the twentieth century, vast shoals of cod found their way to the coastal waters of Greenland and a transition towards commercial fishing, cash economy, and increased urbanization started. After WWII, time was ripe for political change and a reform commission was established by the Danish government, the G-50 Commission. G-50 suggested several changes among which were the further development of a commercial fishing industry and support of the already ongoing centralization of the population [1]. In 1953, the former colony became an integral part of Denmark. During the post-colonial years, profound changes took place in Greenland. The population increased from 24,000 to 56,000 in 2017, and the movement from villages to towns continued. While in 1950, 50% of the population lived in villages; this proportion had decreased to 13% in 2017. Modern houses with running water and bathrooms were built to replace the traditional crowded stone-and-turf houses. Hospitals were built in all towns. Alcohol consumption increased and peaked in 1982 and 1987 with an average consumption of the equivalent of 22 l pure alcohol per adult per year. Greenland became connected internally and to the outside world by commercial airlines and telephone, and in 1992, the introduction of real-time TV further integrated Greenland in the world community. Widespread availability of Internet has improved participation in the global community although the cost of data traffic is still prohibitively high. After G-50, the proportion of migrant workers from Denmark increased from 4 to 19% in 1975. It is now down to 11% [2].

In 2017, the population of Greenland numbered 55,860 of which 90% were born in Greenland and 10% were born outside Greenland, mostly in Denmark. Place of birth is a proxy for ethnicity used by Statistics Greenland and other agencies; for adults living in Greenland, this is a rough but useful estimate of ethnicity as Greenlanders (Inuit) or Danes. Kalaallissut, an Inuit language, is the vernacular spoken by virtually all Greenland Inuit, while Danish is the major second language, spoken by a substantial proportion although far from all.

**Fig. 1:**
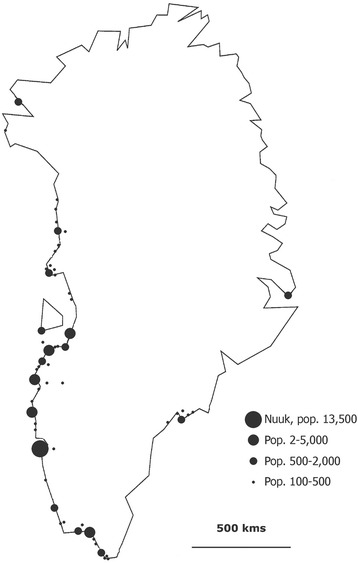
Map of Greenland with communities according to size. Villages with less than 100 inhabitants are not shown

The population is scattered in 17 small towns and approximately 60 villages which are all situated on a narrow coastal strip. A town is defined historically as the largest community in each of the 17 districts. The capital, Nuuk, has 17,000 inhabitants, the second largest town 5600, and the villages have between 500 and less than 50 inhabitants. In the towns are located district school(s), health center or hospital, church, district administration, and main shops. These institutions are absent or present to a much smaller extent in villages. There are no roads connecting the communities. The majority (92%) lives on the West Coast, around 3500 people live on the South East Coast, and about 750 people live in Avanersuaq in the extreme northwest corner of the island (Fig. 1). The communities in the east and extreme north are poorer and less developed than the rest of the country. Countrywide, there are marked socioeconomic and infrastructural differences between towns and villages.

**Fig. 2:**
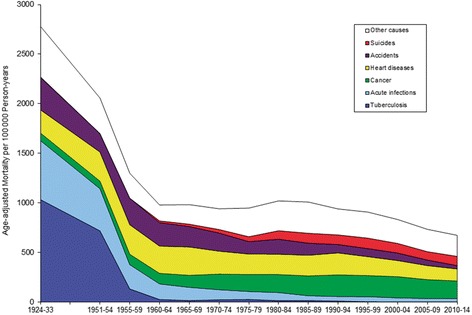
Mortality from selected causes in Greenland 1924–2014. Adjusted for age and sex. Updated from [5, 7]

Based on information from Bertelsen [3], the annual reports of the Chief Medical Officer [4], and the Greenland Registry of Causes of Death, Fig. 2 gives an overview of causes of death in the Inuit population of Greenland since 1924, reflecting the epidemiological transition that the Inuit in Greenland have undergone [5]. Mortality from tuberculosis and acute infectious diseases declined significantly until the 1960s and are now negligible as causes of death. Since 1960, a decrease in mortality from infectious diseases, heart diseases, and accidents has been balanced by an increase in mortality from cancer and suicides. Life expectancy at birth increased from 63 years in 1977–1981 to 71 years in 2011–2015 [2]. It is considerably lower than in Denmark and most European countries but higher than among many other indigenous and tribal peoples [6].

A number of population health surveys as well as public health documents by the Greenland Government have identified alcohol, tobacco, and obesity as main risk factors [7, 8]. The purpose of the paper is to discuss the prevalence trends for these risk factors in post-colonial Greenland and their impact on public health.

## Material and methods

The review is based on published results from studies among the Inuit in Greenland and official statistics. Much of the information is based on two sources, i.e., the Greenland register of causes of death and the recurrent population health surveys conducted by the National Institute of Public Health in Denmark in cooperation with the Ministry of Health in Greenland.

The Greenland Registry of Causes of Death has information on all deaths in Greenland since 1968 coded according to the International Classification of Diseases. The Death Registry currently covers the period 1968–2014 and has information about all 19,776 deaths in Greenland during that period of which 94% have a diagnosis according to ICD-8 or ICD-10. Statistics Greenland has information about demography and import of alcohol and tobacco. Since 1993, regular population health surveys have been carried out. Information is collected by interviews and questionnaires supplemented with clinical examinations and blood analyses [9]. The most recent survey is from 2014. Among the indicators presented here, a few non-standard ones merit further description. Harmful use of alcohol is based on the CAGE questionnaire which is a four-item questionnaire designed to detect alcoholism in a general population [10]. Alcohol problems in childhood home and prevalence of sexual abuse and violence during childhood were self-reported answers to three questions which have been used in the population health surveys in Greenland since 1993. Information on food insecurity was also self-reported and constituted a positive answer to the question “In the past 12 months, were there times when there was no food in your house and no money to buy more?”.

It should be noted that like in the other Nordic countries, the official registers do not have information by ethnicity. In Sweden, for example, this is a severe obstacle to the analysis of health among the indigenous Sami who are a minority in all jurisdictions for which health data is collected [6]. In Greenland, on the other hand, the fact that the Inuit are a vast majority facilitates the interpretation of nationwide data as representing the Inuit. In addition to this, place of birth (in Greenland or outside Greenland) is still an acceptable marker for Inuit ethnicity among adults living in Greenland.

Research papers, reports, and other so-called grey literature have been identified from a comprehensive bibliography (until 1995) [11], PubMed (1996 to present), and our own collection of reports and academic theses from more than 35 years of dedication to the study of public health in Greenland.

## Results

### Alcohol, adverse childhood experiences, and suicide

A high prevalence of misuse of alcohol and hashish is a great public health challenge in Greenland. Alcohol is not only considered a challenge for those who drink but even more so for the family and closely linked to the high prevalence of adverse childhood experiences. More complex social- and mental health issues are often tied to alcohol problems in a family and so is the neglect of children. The typical consumption pattern in Greenland is characterized by weekly or monthly episodes of high consumption, binge drinking, which has multiple health and social risks [12]. The misuse of alcohol is often combined with marijuana and to some extent also problematic gambling behavior [13].

In 1955, the sale of alcohol was permitted to the general population and the import of alcohol, which for all practical purposes in Greenland is equal to the consumption, increased from the equivalent of 6 l of pure alcohol per adult (age 15+) in 1960 to 22 l in 1987 but has decreased steadily since then and is now less than 9 l [14]. Although the import of alcohol has gone down since the 1990s, Greenland still struggles with the consequences of several generations who grew up with alcohol problems in their childhood home [15]. In generations born between 1965 and 1995, as many as 65% reported alcohol problems in their childhood home. In comparison, the prevalence was below 40% for generations born before 1945 [12]. This temporal trend corresponds well with the increase and peak in 1978 in the alcohol import as those born during 1965–1995 are probably reporting their childhood conditions during 1970–2000.

Although the alcohol consumption in Greenlandic is decreasing and it seems that the level of alcohol problems in families is too, the prevalence of harmful use of alcohol was still 21% among men and 22% among women with children living in their household, when measured in the 2014 health survey [12]. Correspondingly, 53% of men and 40% of women with children living in their household reported drinking more than five drinks at one occasion on a monthly basis (binge drinking) in the survey.

Growing up in a home with alcohol problems is closely linked with other common adverse childhood experiences in Greenland, i.e., sexual abuse and violence during childhood, both with long-term effects on children’s and adults’ mental and physical health. Child sexual abuse does not generally happen between close relatives. More often, it is the case that parents are unable to control what is happening in a home steeped in alcohol [16, 17]. In a population health survey from 2014, about one third of the respondents self-reported having been victims of sexual abuse before they turned 18. This number was unchanged from 2005, when it was measured in a previous health survey [12]. Measured as a total, 66% of adults participating in the 2014 Health Survey either reported having experienced alcohol problems in their childhood home, having been a victim of violence or sexual abuse, or a combination of two or more of these adverse childhood experiences (Table 1). The prevalence of different adverse childhood experiences varies according to region and place of residence with the highest prevalence of alcohol problems in childhood home found in the capital of Nuuk vs. in other towns and villages; the highest prevalence of violence in Nuuk and other towns vs. villages; and a higher prevalence of sexual abuse during childhood in East Greenland vs. West Greenland.

**Table 1:** Prevalence of selected adverse childhood experiences: experienced alcohol problems during childhood as well as violence and sexual abuse during childhood by location and region. All three indicators are self-reported by adult participants in health surveys. Unpublished analyses from the Greenland Health Survey 2014 [12]

	Often experienced alcohol problems in childhood home *N* = 1147	Victim of violence during childhood *N* = 1125	Victim of sexual abuse during childhood *N* = 977
	%	%	%
Location			
Nuuk	33.1	50.0	37.1
Towns	21.7	46.7	36.7
Villages	18.2	36.9	39.3
*p*	0.002	0.004	0.74
Region			
West Greenland	21.4	44.2	36.0
East Greenland	23.2	41.5	46.1
*p*	0.62	0.55	0.03

There is a close link between the adverse childhood experiences illustrated above and suicidal behavior with a higher prevalence of suicidal thoughts and attempts among those who experienced sexual abuse and alcohol problems during childhood [15]. The overall rate of suicides in Greenland has been unchanged throughout the past 40 years [18] and is still among the highest in the world [19]. A significant increase took place from 1960 to 1980, and since 1980, the crude suicide rate has been around 100 per 100,000 person-years [18]. Suicides are considerably more common among men than among women. There is a distinct peak in the age group 20–24 for men and 15–19 for women, and suicides are committed at an increasingly younger age in the youngest generations.

Although the overall rate has not changed, the temporal trend of suicides differs among regions [18]. In the towns of West Greenland, there was an increase until the late 1980s followed by stagnation of rates. In contrast, the capital had an early rise in rates in 1980–1984 followed by a decrease, and rates have been lower than in the other towns in West Greenland since 1985–1989. From the start, the suicide rates in the villages in West Greenland were relatively low but a steady increase has brought them at the same level as those of the towns. Finally, the suicide rates in East and North Greenland have remained the highest of all since 1985, recently more than twice as high as rates in West Greenland although there was a decline in the most recent period, possibly artificial due to small absolute numbers. The different temporal patterns suggest that social and economic development influences the suicide pattern. Social and economic development started first in the capital, and socioeconomic conditions have generally become better there than in the rest of the country. In remote East and North Greenland, the development started later, and the improvements in socioeconomic conditions have not yet reached those achieved in the rest of the country.

As many as 60% of the adult population have lost friends and family members to suicide (unpublished results from Inuit Health in Transition Greenland Survey). Among youth aged 15–17, about a third have lost family members and one fifth have lost a close friend or partner to suicide [13]. This puts the whole population at risk because the risk as the risk of dying by suicide is elevated among the bereaved.

There is little evidence from Greenland around interventions in suicide prevention, but studies from Arctic Canada and Alaska suggest a positive influence on youth’s mental wellness from land-based programs with focus on intergenerational relationships, cultural identity, and spending time on the land [20].

### Diet and obesity

Obesity is a major public health challenge in Greenland because of its high and rapidly increasing prevalence and its association with cardiovascular disease, diabetes, and other chronic diseases. Obesity, diet, and physical activity are closely linked. The traditional diet of the Inuit consisted mainly of sea mammals and fish with some local plants such as seaweed and berries. During colonial times, imported food items (grain, dried peas, dried fruit, and sugar) were added to the diet, but around 1900, still 82% of the energy intake came from locally harvested sources [21]. During the twentieth century and especially since 1990, a combination of dietary transition and reduced physical activity has resulted in a rapidly increasing prevalence of obesity.

A number of dietary surveys conducted since 1955 have focused on the proportion of locally harvested food in the diet. Although the methods have been different, there is a clear trend of decreasing consumption of especially sea mammals from 35% in 1955 [22] to 21% in 2007 [23]. Studies from the beginning of the twenty-first century using Food Frequency Questionnaires covering multiple local and imported food items have shown that the intake of a traditional diet increases with age while the adherence to an unhealthy diet (fast food, cakes, sweets, soda pop) decreases with age [24]. A parallel secular trend was also present [12]. Furthermore, traditional food was consumed more often in villages than in towns and imported meat conversely, while there was no variation for an unhealthy dietary pattern. The social variation between hunters/fishermen, unskilled workers, and white-collar employees was similar [24, 25]. Further studies indicate that a reduction of the consumption of sea mammals and fish as well as an increase in the consumption of sugar was predominantly found in the most traditionally living population in the villages (Bjerregaard P, unpublished oral presentation, 2016).

The decreased consumption of traditional food was accompanied by an increased consumption of sugar and saturated fat, but also of fiber, while the intake of the beneficial n-3 fatty acids (“fish oils”) decreased [23]. There are no time series for total dietary energy intake.

Food insecurity has only recently become an issue of scientific study in Greenland. Food insecurity was shown to be closely associated with socioeconomic conditions including region of residence; in remote East Greenland, 20% of participants in a survey in 2014 reported occasional lack of food during a 12-month period due to lack of money compared with 10% in West Greenland and 7% in the capital [12].

The level of total physical activity has decreased since the reduction due to increased mechanization of labor, and domestic chores have not been balanced by an increase in leisure time physical activity [26]. Neither the population health surveys nor other studies offer time series for physical activity.

One study of middle-aged Inuit in East Greenland showed a proportion of obese persons in 1963 (BMI ≥ 30 kg/m^2^) of 2.0% in men and 8.3% in women [27]. With a similar cut point and age group, the figures from the first countrywide population health survey in 1993 were already higher (18% among men and 10% among women) [12]. The population health surveys have monitored the prevalence of obesity in countrywide samples among Inuit of all ages since 1993.

The prevalence of obesity (BMI ≥ 30 kg/m^2^) as well as that of central obesity (waist circumference ≥ 102cm for men and ≥ 88 cm for women) more than doubled from 1993 to 2014. In absolute numbers, obesity increased from 12.6 to 27.3% and central obesity from 23.2 to 47.4%. Women were more often obese than men, especially for central obesity [8]. High and increasing prevalence of obesity has been shown also among the Inuit in Alaska and Northern Canada [28, 29].

In the first surveys during the 1990s, there was no social variation in obesity, but in the two surveys in the new millennium, both general and central obesity showed a statistically significant increasing trend by social position among both men and women [12, 30].

In numerous studies from around the world, obesity is associated with metabolic risk factors such as, for example, 2-h glucose and insulin, blood pressure, triglyceride, and HDL cholesterol. The Inuit in Greenland had lower levels of these risk factors than a population sample from Denmark at any given level of obesity [31]. Similar results were found among the Inuit of Alaska and Northern Canada [28]. This may be due to a relatively higher proportion of subcutaneous than visceral fat among the Inuit [32].

### Smoking

Smoking is a major public health challenge in Greenland because of its high prevalence and related morbidity and mortality from a number of diseases. In a study from Denmark where the smoking prevalence in 2000 was 37% plus 24% ex-smokers, it was estimated that during 1997–2001, smoking was a contributing factor to one quarter of all deaths in addition to a substantial reduction of life expectancy and a high prevalence of chronic diseases [33]. In 2014, 60% of Inuit in Greenland were current smokers and 27% ex-smokers [12]. The proportion of deaths with smoking as a contributing factor is probably correspondingly higher. Lung cancer is the most common cancer in Greenland amounting to 34% of cancer deaths during 2000–2014 (Bjerregaard P, unpublished analysis of the Greenland Register of Causes of Death, 2017).

The import of cigarettes peaked in 1980–1984 at 11.1 cigarettes per person per day and has declined steadily since then [2]. However, the import of cigarette paper has increased considerably which jeopardizes the validity of the cigarette import statistics as a measure of tobacco consumption. In actual fact, the import of cigarettes plus cigarette paper corresponds well with the reported consumption in the population health surveys leaving room for the use of some of the cigarette paper for marijuana cigarettes [34].

The population health surveys from 1993 to 2014 show that the prevalence of smoking is decreasing albeit slowly. There was a clear decrease from 78% in 1993 to 60% in 2014 of daily or occasional smokers, but this was mostly due to a reduction of occasional smoking. Since 1999, the prevalence of daily smoking has remained stable at around 57–59%.

There is a distinct social trend in smoking. The prevalence in 2014 was considerably lower among white-collar employees (35 and 41% among men and women) than among the unemployed (82%). Analyses of data from population health surveys in 2005 and 2014 show that smoking prevalence is lower among Greenlanders with some post-school education and among hunters/fishermen than among blue-collar workers ([8]; Bjerregaard P, unpublished oral presentation, 2017).

## Conclusions

Until the 1950s, life in Greenland was based on fishing and hunting of sea mammals, housing conditions were appalling for the majority of the Inuit, and the disease pattern was characterized by a very high prevalence and mortality of tuberculosis and other infectious diseases. Fifty years of social change and economic development have resulted in an epidemiological transition during which lifestyle-related diseases have replaced acute and chronic infections as the main public health challenges. We have outlined three public health issues of different but major importance among the Inuit in Greenland.

Alcohol import has decreased steadily since a peak in 1978 and is now less than the equivalent of 9 l pure alcohol per adult per year. In Greenland, the negative social effects of excessive alcohol consumption far outweigh the physical damages, and exposure to the disruptive effects of alcohol misuse in the childhood of many Inuit especially during the 1970s and 1980s reaches far into the adult life of these children and into subsequent generations. Research and observations of social welfare workers have indicated suicides, suicidal thoughts, sexual abuse, lack of parental skills, educational problems, and a variety of physical and mental health problems to be associated with exposure to alcohol problems in the childhood home. The epidemiological research is cross-sectional, and the observations of the social welfare workers are anecdotal and hence both are unable to prove causal relationships but there is a strong plausibility. One important lesson from this is that prevention and treatment of alcohol damage must extend over generations and that there is no cause for relaxation or overly rejoicing because the alcohol import statistics show a continuously decreasing trend.

Obesity is on the increase and has been so since the first measurements in the 1960s. This has happened parallel to profound changes in dietary habits and increased mechanization of both work and domestic chores. Recent genetic studies have elucidated the genetic history of the Inuit in Greenland and have discovered genes that put the Inuit at a particular risk of becoming obese and developing diabetes [35–37]. Obesity and its complications such as diabetes, hypertension, and other cardiometabolic diseases together with an aging population threaten the capacity of the health care system in Greenland and has the potential to become a serious threat to the national economy in the twenty-first century. It is thus of the utmost importance to curb the ongoing epidemic of obesity through preventive measures. Along with the general tendency towards obesity, other dietary issues should not be forgotten. A sizeable proportion of the population especially in remote areas of Greenland suffers from food insecurity due to economic constraints and the traditionally consumed marine mammals are polluted with long-range man-made chemicals [38].

Tobacco smoking is another risk factor that is decreasing but which due to its magnitude and slow decrease cannot be ignored. It is still about 60% of both men and women who smoke on a daily basis, many more than in all European countries [39]. A reduction in reported smoking was especially seen between 1993 and 1999, whereas the decrease has been negligible during the 15 years from 1999 to 2014.

The diseases and conditions outlined above all contribute towards a low life expectancy at birth—69 years for men and 74 years for women in 2011–2015 [2]—compared with 78 and 84 years for men and women, respectively, on average in the European countries and even lower than in Bulgaria which has the lowest life expectancy at birth in Europe [39].

The Government of Greenland has focused on these public health issues among other things through its public health preventive programs Inuuneritta I (2007–2012) and Inuuneritta II (2013–2019). Inuuneritta I covered nine topics including (1) alcohol and hashish, (2) violence and sexual assaults, (3) suicides, (4) diet and physical activity, (5) reproductive health, (6) smoking, (7) children and adolescents, (8) the elderly, and (9) dental health care [40]. Based on the experience from the first program, it was decided to concentrate on four topics in Inuuneritta II: alcohol and hashish, smoking, physical activity, and diet. Children, adolescents, and families were designated target groups for intervention and health promotion. Social inequality in health is an overarching theme [8]. Inuuneritta is implemented in cooperation among the Government, municipalities, and private organizations. Each topic was designated a year with extra resources for projects and intervention during the period. In 2013, there was countrywide focus on alcohol and marijuana, in 2014 on smoking, in 2015 on physical activity, and in 2016 on diet. In 2018, data will be collected on selected health indicators for the program through countrywide population health surveys among adults, adolescents, and school children, and in 2019, the program will be evaluated among other things based on the progress of indicators. It is yet too early to tell if the targets have been met. The midterm evaluations of both programs [41, 42] concluded that although the implementation is systematic at the government level, there is insufficient translation of the programs from central to local level, which challenges the implementation of the program objectives. We agree with the conclusions of the midterm evaluation that in order to sustainably improve the implementation process of the public health program in Greenland, substantial changes should be made including (1) politically ensuring intersectoral collaboration, (2) reinforcing leadership, (3) improving and sustaining local capacity, and (4) ensuring participation of key stakeholders. Most importantly, the central strategies must be developed with respect to and in dialog with local stakeholders according to community capacities which differ among regions. Improvement of public health is a long-term task, and it is hoped that the public health preventive programs will continue beyond 2019 with an increased focus on the implementation of the good intentions.
